# Quantitative and Qualitative Changes in Diet Associated with the Consumption of Ultra-Processed Foods: A Survey on a Representative Sample of Manufacturing Workers in Brazil

**DOI:** 10.3390/nu15133009

**Published:** 2023-07-01

**Authors:** Raiane M. Costa, Ingrid W. L. Bezerra, Anissa M. Souza, Karina G. Torres, Gabriela S. Pereira, Célia M. M. Morais, Antonio G. Oliveira

**Affiliations:** 1 Postgraduate Program in Health Sciences, Centro de Ciências da Saúde, Universidade Federal do Rio Grande do Norte, Natal 59012-570, Brazil; raianemedeiros@hotmail.com (R.M.C.); anissasouza@hotmail.com (A.M.S.); karinagomestorres@gmail.com (K.G.T.); gaabsp@gmail.com (G.S.P.); 2 Nutrition Department, Centro de Ciências da Saúde, Universidade Federal do Rio Grande do Norte, Natal 59078-970, Brazil; celiamarcia@uol.com.br; 3 Pharmacy Department, Centro de Ciências da Saúde, Universidade Federal do Rio Grande do Norte, Natal 59012-570, Brazil; oliveira.amg@gmail.com

**Keywords:** ultra-processed food, food consumption, nutrient intake

## Abstract

The increasing intake of ultra-processed (UP) foods is causing changes in the profile of food and nutrient consumption, negatively influencing consumer behavior. The present study aimed to evaluate the influence of UP foods on the diet of Brazilian adults, verifying the association between its increasing contribution to total energy and trends in the consumption of other NOVA groups, food subgroups, energy consumption, and macro- and micronutrients. We conducted an observational, cross-sectional study of a probability sample of 921 manufacturing workers in the state of Rio Grande do Norte, Brazil, from a combined stratified and two-stage survey. Trends in consumption across quintiles of UP food contribution to the total energy intake were tested with linear regression. The results showed that higher UP food consumption is associated with a higher intake of energy, carbohydrates and total-, mono- and polyunsaturated fats, saturated fats and trans fats, and the micronutrients calcium, iron and thiamine; and higher consumption of ready-to-eat foods, accompanied by lower consumption of foods that require preparation, such as beans, tubers and roots, vegetables and fruits, which may represent a risk for the development of non-transmissible chronic diseases in this population.

## 1. Introduction

Ultra-processed (UP) foods and products are industrial formulations of substances derived from foods, with little or no whole food, added to a set of cosmetic additives in order to make them attractive, palatable and, consequently, profitable. The ingredients and processes used in the manufacture of these foods are, for the most part, for the exclusive use of the food industry [1], typically consisting of high-energy products, rich in sugar, unhealthy fats and sodium, and low in dietary fiber, proteins, vitamins and minerals [2,3].

Currently, UP foods are part of a classification that categorizes foods according to processing levels: the NOVA classification. Widely used in research related to food consumption, the NOVA classification groups all foods and food products into four categories: in natura or minimally processed, processed culinary ingredients, processed, and ultra-processed [4,5]. The differentiation between the categories considers the type, extent and purpose of the industrial processing they undergo, as well as the physical, biological and chemical processes applied to fresh foods, modifying them according to the interests of the food industry.

The access and excessive intake of UP foods, especially in recent decades, have been causing changes in the profile of food and nutrient consumption, influencing the behavior of consumers and their food choices and modifying the nutritional profile of the food consumed [4,6].

Worldwide statistics on UP food sales show that these products dominate the food supply of high-income countries, and their consumption is now rapidly increasing in middle-income countries [7,8]. In the United Kingdom, a cross-sectional study of about 9000 adults and children, using food consumption data obtained through a four-day food diary, reported a contribution of 56.8% of UP foods to the total daily energy consumption [9]. In Canada, a study of more than 33,000 individuals (age ≥ 2 years) evaluated food consumption using 24-h dietary recall (24HR) and found that the contribution of UP foods to the daily energy intake was 47.7% [2]. In Australia, a cross-sectional study with more than 12,000 individuals (≥2 years), also using 24HR, found that the contribution of UP to daily calories was 42% [10].

Research conducted in different populations showed that UP food consumption (measured by its percent contribution to total energy intake) is inversely related to the nutritional quality of the diet [11]. Evidence from analyses of nationally representative datasets from 11 countries has shown that the substitution of in natura and minimally processed foods for ultra-processed foods was consistently associated with the loss of nutritional quality of diets [11]. Additionally, a systematic review of studies conducted in adult populations from several countries found that UP food consumption was consistently associated with the incidence of chronic noncommunicable diseases (NCDs), including obesity, high blood pressure, diabetes, cardiovascular disease, cancer, and depression [12].

Therefore, considering the implications of UP food consumption on the diet of individuals and its consequences on public health, as well as the paucity in the literature of studies that analyze the effects of UP food consumption on the consumption of other classes and food groups, this study aimed to investigate and describe how an increasing tendency to consume UP food may be accompanied by a trend to consume certain food groups and nutrients. The main study question was whether there is a tendency for unhealthy feeding habits with increasing consumption of UP. Our interest resides in manufacturing workers, who are a relevant segment of the population, in most countries representing over 10% of the active population and being responsible for over 10% of overall national output [13]; therefore, the survey was conducted in a representative sample of that population.

## 2. Materials and Methods

### 2.1. Sampling Plan

This was an observational, cross-sectional study, based on a representative, probability sample of manufacturing workers in the state of Rio Grande do Norte, Brazil. The study was carried out in accordance with the Declaration of Helsinki and approved by the Ethics Committee of the Hospital Universitário Onofre Lopes (No. 2.198.545/2017).

The sampling plan was a combined stratified proportional and multi-stage sample. The strata were the industry size, categorized as small (less than 50 workers), medium (from 50 to 500 workers), and large (more than 500 workers), and the sector of activity. The sectors considered were those with the greatest implementation in the state: food and beverages, non-metallic minerals, and textiles. In the first sampling stage, manufacturing companies were randomly selected in each combination of strata in a number proportional to the total number of companies in the state in that combination of strata, using as a sampling grid of the register of companies maintained by FIERN, the state industry federation. In the second stage, from each company recruited in the first stage, a fixed number of workers were selected by simple random sampling, using a computer-based random number generator, from lists of workers from each company provided by human resource departments.

### 2.2. Population

All companies in the state of the three mentioned sectors of activity that agreed to participate in the research were eligible for the study. The inclusion criteria for the workers were: age over 18 years old, with an effective labor link to the company, who regularly used the cafeteria at lunch, and who gave written consent to participate in the research. Exclusion criteria were pregnant women, temporary employees, interns, and employees on a probationary period.

### 2.3. Study Plan

The selected workers were approached at lunchtime and asked to participate in the survey. After signing the informed consent form, two questionnaires were applied: one to record biodemographic data (age, sex, marital status, education, income, training in the company); and another for recording information on food consumption with the 24 h dietary recall method (24HR).Dietary data recorded from study participants included all consumption in the 24 h prior to the interview, including preparation and cooking methods, ingredients, quantities (in household measures), and the time and place the meal was consumed. In order for the result of the 24HR to reflect the usual food consumption, data were collected between Tuesdays and Saturdays, thus excluding consumption during the weekend.

### 2.4. Characterization of Food Consumption

Information on food consumption obtained as household measures was quantified as units of weight and volume, using previously established references (direct weighing of food, photographic records and specific manuals) for this purpose [14]. The analysis of the nutritional composition of the workers’ food consumption was carried out using reference Food Composition Tables [15,16,17], supplemented, when necessary, with information from food labels. The foods recorded in the 24HR were classified in two ways: (1) according to the NOVA group; (2) according to the food group, following the classification of food groups in the Food Guide for the Brazilian Population, which considers biological, physical–chemical, organoleptic and dietary characteristics, aggregating foods into sets that have similar culinary uses and nutritional profiles [4,18]. Given the variety of foods and preparations referred to in the 24HR, in the present study, the sets aggregated by the Food Guide were broken down into 44 food groups. Most freshly prepared foods, which include items from various food groups, were described in their ingredients, defining the quantities based on a Technical Preparation Sheet. A small number of freshly prepared mixed foods, which are mainly based on unprocessed and/or minimally processed foods, and which are typical of Brazilian cuisine, were classified in the food group with the highest contributing ingredient. More details on the criteria used for this classification were published in another study [19].

### 2.5. Statistical Analysis

The Stata 15.1 statistical program (Stata Corporation, College Station, TX, USA) was used for the statistical analysis of the data. The statistical analysis considered the stratification by company size and activity sector, the two-stage sampling plan with companies as the primary sampling unit and with workers nested within companies, and included sampling weights and correction for the finite population in the calculations. A *p*-value < 0.05 was considered evidence of statistical significance.

The results are presented by food and by nutrient as the mean and standard error of the mean of total consumption, or as the mean per quintile of the percent contribution of UP food to the total energy consumption. To assess trends in the average worker characteristics, nutrient intake, and food consumption across quintiles of UP food consumption, linear regression or logistic regression was used as appropriate.

## 3. Results

The survey was conducted from September 2017 to July 2018. The total study sample consisted of 921 workers from 33 manufacturing industries located in the state of Rio Grande do Norte, Brazil. Thirteen small, fourteen medium and six large companies were included. Regarding the sector of activity, 14 were from the food and beverage sector, 6 from non-metallic minerals, and 13 from the textile sector.

Table 1 presents the population estimates of the mean values of the characteristics of industry workers according to the quintiles of the percentage of kcal consumed in UP relative to the total energy consumed. There was a trend towards lower UP food consumption with age. No association was found between the other characteristics analyzed and the consumption of ultra-processed foods.

In Table 2, we describe the estimates, for the population of manufacturing workers, of the mean consumption of UP. The table details, for each NOVA category (food processing level) and for each food group within each of those categories, the total energy consumed daily and the percentage contribution to the total energy consumed. Minimally processed and in natura foods represented the highest percentage in the average diet (56%), with the main contributors being white meat, rice, red meat, corn and beans. The ultra-processed products had the second highest percentage (19.2%), with the highest contribution from the biscuits, processed meats, and UP breads (toasted, sweet, cheese, hot dog and burger bread) groups. Processed foods represented 16.8% of the total energy intake, mainly from the bread (white) and cheese (mozzarella, curd and butter cheese) groups. Culinary ingredients had the lowest percentage, with a higher expression of consumption specifically of butter (dairy products group contributor to this NOVA category) which was substantially higher than the others (61.1%).

To investigate whether UP food consumption may modify the nutrient content of the diet, the association between the amount of UP food consumption and macro- and micronutrient consumption was evaluated by testing the linear trend between quintiles of consumption of UP food and consumption of each nutrient. Table 3 presents the average daily consumption of nutrients according to the quintiles of the percentage contribution of ultra-processed foods to the total daily energy consumption. Higher UP food consumption was accompanied by a trend towards increased consumption of energy (*p* = 0.010), carbohydrates (*p* = 0.023), total fat (*p* < 0.001), saturated fat (*p* = 0.001), mono (*p* = 0.001) and polyunsaturated fat (*p* =0.025), trans fat (*p* < 0.001) and omega 6 fatty acids (*p* = 0.026). Among micronutrients, higher UP food consumption was associated with a trend towards a higher iron (*p* = 0.020) and calcium (*p* = 0.015) intake. In vitamins, only thiamine showed a trend towards higher consumption with higher UP food consumption (*p* < 0.001).

We also investigated whether UP food consumption might be associated with changes in the qualitative composition of the diet by testing a linear relationship between UP food consumption and the consumption of the food groups described in the Methods section. Table 4 presents the consumption of food groups by quintiles of UP food contribution to total energy intake. Higher UP food consumption is associated with a trend towards greater consumption of biscuits, processed meats, sugary drinks, fast foods, filled biscuits, and sweets and desserts, and a trend towards lower consumption of beans, tubers and roots, vegetables, natural spices, fruits, red and white meat, eggs, vegetable oils, and salt.

## 4. Discussion

The results of this study on manufacturing workers showed that the food consumption of this population has a greater contribution of in natura or minimally processed foods, followed by UP. The results show that higher UP food consumption is associated with a higher intake of energy, carbohydrates and total, mono- and polyunsaturated, saturated and trans fats, and the micronutrients calcium, iron and thiamine. In the food groups, a trend was observed towards greater consumption of ready-to-eat foods, such as cookies, processed meats, sugary drinks, fast foods, sweets and desserts, accompanied by lower consumption of foods that require preparation, such as beans, tubers and roots, vegetables, fruits, meats, eggs and vegetable oils.

The results of this study on the energy distribution of the NOVA categories are in line with the results of a cross-sectional study with data from the Brazilian Household Budget Survey (HBS), with food consumption obtained from two food records in about 32,000 Brazilian individuals over 10 years old, who reported that in natura or minimally processed foods represented 58.1% of the total energy intake, followed by UP food with 20.4% [3].

However, even though there is a predominance of fresh or lightly processed foods in the Brazilian diet, an increase in UP food consumption in the country over the years has also been observed. According to the same HBS, the participation of UP food consumption in the total energy of the diet increased by 44% between 2003 and 2018. In the same period, the participation of in natura and minimally processed foods was reduced by 7%, making clear the gradual replacement by UP food that has been taking place [20,21].

Data from the literature seem to indicate that UP food industries no longer show growth in consumption among high-income countries, which already have high consumption, and are now moving into new markets among low- and middle-income countries in Latin America [7]. As an example, a survey in Chile of 4920 adults and children, using data from the Encuesta Nacional de Consumo Alimentario and evaluating food intake with 24HR, found that the average dietary contribution of UP food in the general population was 28.6%, and that when the contribution of UP foods to the total energy intake increased, the contribution of other NOVA categories and most food groups within these categories decreased [22].

Furthermore, countries with higher-income populations have a considerably higher mean UP food consumption [2,9,10]. Studies in these countries have shown that higher consumption of UP foods is related to a higher consumption of carbohydrates, total fats, saturated fats, trans fats and sodium, and a lower consumption of proteins, dietary fiber, potassium, and other vitamins and minerals [2,9,10,23]. Taken together, those results indicate a high consumption of UP food in high-income populations, and the high participation of UP food in the daily intake of energy and, consequently, of nutrients, with a marked influence on the nutritional composition of the diet.

Regarding food consumption according to the classification by food groups, studies have shown that, with high UP food consumption, the most consumed food groups were soft drinks and sweetened beverages, types of fast foods, packaged salty snacks, confectionery products and ready-to-eat meals. Additionally, the studies also showed a tendency for the other NOVA categories to decrease with the increase in UP food consumption [2,9,10,22,23].

As for micronutrients, the present study shows that with the high consumption of UP foods, there was a statistically significant increase in the consumption of calcium, iron and thiamine. An explanation for these findings may lie in the industry’s nutritional strategies, such as food fortification, reformulation, and functionalization, which are being used to advocate and market their highly processed foods and products [24,25].

However, these strategies are detrimental to public health efforts aimed at improving diet quality by promoting the consumption of fresh and minimally processed foods [24,25,26,27]. There are many public health-oriented mandatory fortification policies to address micronutrient deficiencies [24,25]. For example, in Brazil, mandatory iron fortification was implemented in wheat and corn flour, which are basic ingredients for numerous ultra-processed products [28]. Along with these mandatory fortification programs, food manufacturers adopt discretionary and random fortification of micronutrients in their food products, using it as positive marketing to attract consumers [24,29]. However, the reformulation/fortification of these products does not modify their classification as UP, nor does it influence other mechanisms through which these dietary patterns impact health, therefore not eliminating the risks of inadequate nutrition associated with their consumption [24,30].

In relation to the increased consumption of calcium and iron with increased UP food observed in this study, a study carried out in the city of Pelotas, Brazil, with more than 4000 young adults, assessing food consumption using the food frequency questionnaire and estimating the daily proportion of energy intake and macro- and micronutrients attributed to UP foods, observed that the proportion of individuals with adequate consumption of iron and calcium was higher among those with higher UP food intake, both due to the presence of cheese and yogurt in food consumption, as well as the use of milk and its derivatives as ingredients of several UP food products [31]. The results of the present study also showed a tendency towards an increase in mono- and polyunsaturated fats with the increase in the contribution of UP foods to the total daily energy consumption, which can be explained by the presence of vegetable oils in the list of ingredients of ultra-processed products, such as palm oil, widely used by the food industry and a source of this type of fat [32].

In view of the results, we conclude that a higher consumption of UP foods is associated with a higher intake of energy, carbohydrates and fats, from a greater consumption of ready-to-eat foods to the detriment of foods in their most natural form. The implications of these findings for the definition of public policies may be either that the effect of UP food consumption on human health may be aggravated by a trend in the preference for unhealthy foods, or that UP food consumption may mediate the effect of unhealthy feeding habits on the development of non-transmissible chronic diseases. In either case, future studies on the effects of UP food on human health should consider the possibility of joint effects of food preferences and/or macronutrient consumption, which may represent a risk to the medium- and long-term development of non-transmissible chronic diseases in this population.

This study has some limitations, such as the survey being limited to one federate state in the country and restricted to manufacturing workers as well as the use of the 24HR, which, despite being a survey widely used in epidemiological studies, requires the memory of the interviewee to access reliable information on food consumption. However, the size of the survey, the representativeness of the sample, and the total representation of the state (which has geographic and economic similarities with the other states in the region), are aspects of the study methodology that contribute to the robustness of the results.

## Figures and Tables

**Table 1:** Population characteristics according to quintiles of the contribution of ultra-processed foods to total energy intake.

Subject Variable	Overall		Quintiles of Consumption of Ultra-Processed Foods (% of Total Energy Intake)					*p*-Value
	Mean/%	S.E.	Q1	Q2	Q3	Q4	Q5	
Age (yrs)	38.3	0.50	40.5	40.2	38.2	38.0	35.1	0.002
Male (%)	55.8	1.96	53.3	57.7	57.0	55.0	56.3	0.87
Married (%)	62.8	2.41	63.8	67.6	68.5	59.3	56.7	0.20
Children	1.37	0.06	1.55	1.37	1.29	1.44	1.22	0.20
Education (yrs)	10.9	15.7	10.8	10.3	11.5	10.9	10.9	0.56
Income (minimum wages)	1.55	0.10	1.58	1.42	1.89	1.31	1.62	0.86
In-house formation (%)	19.3	1.92	17.9	11.4	28.8	19.3	18.2	0.58
BMI (kg/m^2^)	27.5	0.25	28.3	26.6	27.2	26.8	28.5	0.80
Waist circumference (cm)	90.3	0.60	92.2	89.1	90.2	88.6	91.7	0.72

Means and percentages are population estimates and S.E. is the standard error of the means.

**Table 2:** Energy intake by NOVA categories and food groups within categories.

NOVA Categories	Absolute Intake (Kcal/day)		Relative Intake (% of Total Energy Intake)	
	Mean	S.E.	Mean	S.E.
Unprocessed or minimally processed food	1107.9	24.0	56.0	0.92
White meat	171.2	8.00	9.04	0.48
Rice	138.5	7.52	7.26	0.36
Red meat	115.8	9.47	5.62	0.46
Corn	114.1	8.00	5.25	0.37
Beans	103.6	4.29	5.28	0.22
Tubers and roots	99.4	9.45	4.98	0.47
Pasta	85.0	6.62	3.69	0.26
**Milk and Dairy (whole, reduced-fat, and low fat milk as powder or liquid)**	62.2	4.72	3.15	0.24
Eggs	53.3	5.26	2.51	0.23
Fruits	50.0	3.44	3.05	0.24
Vegetables	20.7	1.00	1.10	0.06
Soups	20.1	2.56	1.29	0.19
Coffee and tea	20.0	0.80	1.03	0.04
Fruit juices	17.4	1.13	0.85	0.06
Natural spices	5.64	0.32	0.28	0.03
Oilseeds	3.79	1.46	0.24	0.10
Leafy vegetables	0.75	0.10	0.04	0.01
**Processed culinary ingredientes**	**168.3**	**5.08**	**8.04**	**0.19**
Added sugar	91.2	3.27	4.36	0.13
Plant oils	63.7	2.77	3.06	0.12
Milk and Dairy (butter)	13.2	1.97	61.1	0.08
**Processed foods**	**375.5**	**19.4**	**16.8**	**0.70**
Breads (white bread)	138.1	8.67	6.52	0.40
**Milk and dairy (mozzarella, curd, and butter cheese)**	50.1	7.41	2.31	0.31
Red meat	46.9	6.51	2.03	0.24
Cakes	41.6	6.67	1.70	0.24
Fried and baked snacks	31.4	6.16	1.39	0.27
Tubers and roots	22.0	3.41	1.00	0.16
Sweets and desserts	18.0	2.88	0.80	0.13
Biscuits	3.51	3.20	0.15	0.13
“Feijoada”	1.16	0.99	0.02	0.02
White meat	0.95	0.50	0.04	0.02
**Ultra-processed foods**	**404.6**	**20.7**	**19.2**	**0.89**
Biscuits	102.9	10.5	4.88	0.46
Processed meats	59.3	6.01	2.85	0.30
**Breads (toasted, sweet, cheese, hot dog, and burger)**	42.2	6.16	2.08	0.33
Sweets and desserts	27.9	4.81	1.16	0.19
Fried and baked snacks	25.6	5.14	1.08	0.23
Margarine	24.6	2.65	1.14	0.13
Pasta	21.1	5.12	1.20	0.33
Fast Foods	19.0	5.58	0.90	0.27
Sugary drinks	14.9	2.47	0.63	0.10
Whole grains	13.8	3.47	0.95	0.27
Stuffed biscuits	9.91	3.15	0.42	0.12
Dietetic supplements	9.86	6.56	0.46	0.31
**Milk and Dairy (Yogurt, petit Suisse, dairy mixtures, dairy drinks, chocolate milk)**	9.03	1.88	0.47	0.09
White meat	8.05	5.73	0.23	0.13
Snacks	2.52	1.60	0.10	0.06
Granola	2.12	1.07	0.10	0.05
Red meat	2.10	0.69	0.13	0.06
Vegetables	0.33	0.20	0.01	0.01
Tubers and roots	0.17	0.17	0.00	0.00

Means are estimates of population means and S.E. is the standard error of the means.

**Table 3:** Mean dietary content of nutrients in the diet according to the dietary share of ultra-processed foods.

Nutrient	Quintile of Consumption of Ultra-Processed Foods in the Diet (% of Total Energy Intake)					*p*
	Q1	Q2	Q3	Q4	Q5	
	Mean	Mean	Mean	Mean	Mean	
Energy intake	1767.6	2018.0	2143.9	2122.9	2179.0	0.010
Proteins (g)	96.7	102.9	103.7	98.7	92.7	0.42
Fats (g)	48.8	58.6	67.4	67.0	73.7	<0.001
Carbohydrates (g)	233.1	269.0	279.4	280.0	282.1	0.023
Dietary fiber (g)	26.3	25.8	27.8	25.4	23.1	0.17
Sodium	4081.0	4290.7	4781.6	4535.3	4575.2	0.13
Saturated fats (g)	16.5	19.1	22.7	22.5	22.9	0.001
Ca (mg)	384.9	459.4	521.2	498.0	563.4	0.015
P (mg)	1142.4	1266.7	1231.8	1226.2	1225.1	0.58
Mg (mg)	228.3	234.7	255.9	241.8	282.2	0.12
Mn (mg)	2.21	2.20	2.84	2.53	5.49	0.08
Fe (mg)	9.04	9.63	10.2	11.7	13.3	0.020
K (mg)	2286.6	2361.9	2404.6	2394.0	2329.9	0.76
Cu (mg)	2.44	1.73	1.23	1.06	0.93	0.06
Zn (mg)	10.8	11.6	12.2	11.6	11.8	0.55
Vitamin A (mcg)	1614.2	1224.3	602.8	431.3	342.2	0.06
Thiamin (mg)	0.86	1.12	1.14	1.16	1.66	<0.001
Riboflavin (mg)	1.20	1.32	1.23	1.18	1.26	0.94
Vitamin B6 (mg)	0.80	0.94	0.97	0.91	1.09	0.20
Niacin (mg)	19.5	21.6	18.6	20.4	23.4	0.27
Vitamin C (mg)	134.3	212.2	116.7	144.3	147.9	0.72
Se (mg)	83.6	85.3	81.5	84.5	91.7	0.49
Vitamin D (mg)	2.23	3.53	2.08	2.59	3.09	0.58
Vitamin E (mg)	4.48	4.68	4.34	4.40	7.05	0.25
Cobalamin (mg)	7.93	8.54	5.67	8.04	4.10	0.23
Monounsaturated fat(g)	14.3	17.4	20.1	19.8	20.6	0.001
Polyunsaturated fat (g)	11.0	13.5	16.4	14.3	14.7	0.025
Omega 3 (g)	1.10	1.40	1.40	1.77	1.27	0.24
Omega 6 (g)	9.52	11.7	12.5	12.5	12.5	0.026
Trans fat (g)	1.33	1.73	2.07	2.70	3.03	<0.001

The values referring to the average estimates of macronutrients and micronutrients were obtained from the nutritional analysis of individual intakes, using the food composition tables as a reference.

**Table 4:** Food consumption according to the proportion of ultra-processed food in the diet of workers.

Food Groups	Quintiles of the Contribution of Ultra-Processed Foods in the Diet (% of Total Energy Intake)					*p*
	Q1	Q2	Q3	Q4	Q5	
	Mean	Mean	Mean	Mean	Mean	
Beans	159.8	146.3	141.7	134.6	107.3	0.014
Rice	121.4	108.5	106.8	120.6	82.6	0.11
Corn	89.7	121.6	130.0	78.8	75.5	0.15
Whole grains	5.49	7.16	15.3	10.7	11.9	0.23
Breads	51.8	63.9	64.1	70.1	45.2	0.77
Pasta	52.4	61.1	64.4	71.9	81.6	0.06
Biscuits	0.36	6.56	17.8	30.2	60.3	<0.001
Tubers and roots	78.0	110.0	57.9	60.7	31.1	0.010
Leafy vegetables	4.85	2.92	3.58	4.82	5.31	0.52
Vegetables	72.2	53.5	60.3	50.4	41.7	0.004
Soups	72.8	46.3	79.2	69.4	71.4	0.70
Natural spices	10.9	10.9	11.1	11.0	7.63	0.016
Industrialized condiments	4.91	7.36	6.34	6.17	4.30	0.41
Fruits	95.1	93.6	92.7	86.8	52.5	0.052
Fruit juices	230.4	234.2	210.7	186.3	182.9	0.20
Oilseeds	0.59	1.38	1.21	0.15	0.12	0.11
Milk and dairy	86.0	107.2	120.9	98.9	98.8	0.75
Red meat	65.2	74.3	90.5	71.1	37.2	0.046
White meat	121.9	109.3	91.6	94.5	87.3	0.051
Eggs	33.6	37.2	18.0	25.8	10.0	0.006
Processed meats	0.43	11.2	20.8	21.9	38.4	<0.001
Sugary drinks	27.3	64.9	55.4	99.4	89.9	0.004
Coffee and tea	177.5	204.4	212.3	237.7	211.6	0.11
Fast Foods	0.00	0.00	2.33	6.60	52.3	0.006
Fried and baked snacks	9.91	9.42	28.2	11.9	25.8	0.08
Stuffed biscuits	0.17	0.19	0.19	2.94	5.90	0.024
Sweets and desserts	4.87	16.8	14.5	27.2	38.3	0.005
Added sugar	21.4	22.7	22.0	24.1	22.1	0.65
Margarine	1.23	3.60	3.44	3.94	2.96	0.40
Plant oils	7.55	7.59	7.63	7.33	5.10	0.04
Salt (g)	6.32	5.85	6.00	5.54	4.34	0.001
Tripe/chitterlings	16.3	7.02	4.07	1.48	0.08	0.06
Cakes	12.2	9.92	9.35	14.3	8.99	0.89

## Data Availability

Study data are available upon reasonable request to the corresponding author.
